# Leveraging Reconfigurable Massive MIMO Antenna Arrays for Enhanced Wireless Connectivity in Biomedical IoT Applications

**DOI:** 10.3390/s25185709

**Published:** 2025-09-12

**Authors:** Sunday Enahoro, Sunday Cookey Ekpo, Yasir Al-Yasir, Mfonobong Uko

**Affiliations:** 1 Communication and Space Systems Engineering Research Team, Manchester Metropolitan University, Manchester M1 5GD, UK; sunday.enahoro@stu.mmu.ac.uk (S.E.); s.ekpo@mmu.ac.uk (S.C.E.);; 2 Department of Communication and Informatics Engineering, Al-Farqadein University College, Basrah 61004, Iraq

**Keywords:** reconfigurable MIMO antennas, Bio-IoT, smart healthcare, hybrid beamforming, adaptive signal processing, wearable sensors, energy efficiency, mmWave communication, sub-6 GHz, interference mitigation, Wireless Body Area Network (WBAN), biomedical applications, robust beamforming, machine learning, patient mobility, 5G/6G healthcare networks

## Abstract

The increasing demand for real-time, energy-efficient, and interference-resilient communication in smart healthcare environments has intensified interest in Biomedical Internet of Things (Bio-IoT) systems. However, ensuring reliable wireless connectivity for wearable and implantable biomedical sensors remains a challenge due to mobility, latency sensitivity, power constraints, and multi-user interference. This paper addresses these issues by proposing a reconfigurable massive multiple-input multiple-output (MIMO) antenna architecture, incorporating hybrid analog–digital beamforming and adaptive signal processing. The methodology combines conventional algorithms—such as Least Mean Square (LMS), Zero-Forcing (ZF), and Minimum Variance Distortionless Response (MVDR)—with a novel mobility-aware beamforming scheme. System-level simulations under realistic channel models (Rayleigh, Rician, 3GPP UMa) evaluate signal-to-interference-plus-noise ratio (SINR), bit error rate (BER), energy efficiency, outage probability, and fairness index across varying user loads and mobility scenarios. Results show that the proposed hybrid beamforming system consistently outperforms benchmarks, achieving up to 35% higher throughput, a 65% reduction in packet drop rate, and sub-10 ms latency even under high-mobility conditions. Beam pattern analysis confirms robust nulling of interference and dynamic lobe steering. This architecture is well-suited for next-generation Bio-IoT deployments in smart hospitals, enabling secure, adaptive, and power-aware connectivity for critical healthcare monitoring applications.

## 1. Introduction

The rapid expansion of the Internet of Medical Things (IoMT) has revolutionized how healthcare services are delivered, enabling real-time patient monitoring, remote diagnostics, and pervasive sensing using wearable and implantable devices [1,2]. These medical-grade devices require reliable, low-latency, and interference-robust wireless connectivity to support critical applications in smart hospitals, elderly care, and home-based healthcare environments. However, the increasing density of biomedical devices and the need for guaranteed Quality of Service (QoS) introduce significant challenges in wireless channel reliability, interference suppression, and energy efficiency [3,4].

Massive multiple-input multiple-output (MIMO) systems, equipped with a large number of antenna elements at the base station (BS), offer promising solutions by spatially multiplexing users and enhancing spectral efficiency [5,6,7]. Reconfigurable massive MIMO extends this capability by enabling dynamic adaptation of the antenna configuration to suit varying user loads, channel conditions, and mobility profiles [8]. These features are particularly beneficial for Biomedical Internet of Things (Bio-IoT) applications, where users (e.g., of wearable ECG sensors or implanted glucose monitors) exhibit diverse movement patterns and channel requirements.

Among separate hardware-focused contributions, ref. [9] delivered several reconfigurable RF front-end designs integral to dynamic Bio-IoT connectivity. Their work presented the Highly Adaptive Reconfigurable RF Front-End (HARRF) that intelligently switches between sub-6 GHz and satellite frequency bands using a switchable SPDT matching network, enabling automatic adaptation to fading or congestion. Another study integrated multi-source RF–perovskite energy harvesting with hybrid MIMO interfaces, enhancing sustainable power use while maintaining beamforming performance [10]. These combined software–hardware innovations provide the foundation for fully reconfigurable massive MIMO systems optimized for biomedical applications.

Other recent works have also demonstrated hybrid analog–digital beamforming as an efficient approach to reducing hardware complexity and energy consumption while maintaining high performance in mmWave and sub-6 GHz systems [8,11]. This approach is particularly suitable for wearable Bio-IoT deployments where power and size constraints are critical [12]. Furthermore, beamforming strategies such as Zero-Forcing (ZF), Minimum Variance Distortionless Response (MVDR), and Recursive Least Squares (RLS) have been explored to mitigate inter-user and intra-body interference [13,14,15,16,17,18,19].

Despite these advancements, few studies have comprehensively addressed the integration of reconfigurable massive MIMO with QoS-aware beamforming specifically for Bio-IoT scenarios in smart healthcare facilities. In this work, we propose a system-level architecture that leverages reconfigurable antenna arrays and hybrid beamforming to ensure ultra-reliable, low-latency connectivity across diverse biomedical devices. We also incorporate realistic sub-6 GHz urban macrocell channel models (3GPP UMa), BPSK/QPSK modulation, and adaptive beam patterning tailored to user mobility.

Although some simulation cases use relatively small configurations ($N_{t}$ = 4–32) for tractability and benchmarking, our framework is extended up to $N_{t}=128$ antennas. This scale is consistent with the widely accepted definition of massive MIMO in sub-6 GHz and mmWave systems, where tens to hundreds of antennas are already regarded as massive due to their ability to provide high spatial multiplexing and interference suppression gains [7,20,21].

While this study primarily employs system-level simulations, we also outline an ongoing hardware-in-the-loop testbed implementation using an NI USRP E310 and TMYTEK BBox phased array to validate the architecture under practical impairments such as analog non-linearities and synchronization drift.

Building upon foundational research in antenna reconfiguration [22] and practical wireless health monitoring systems [23], our contribution includes a comparative analysis of classical and advanced beamforming algorithms across multiple performance metrics including BER, SINR, latency, jitter, energy efficiency, and throughput under varying antenna counts and user loads.

In contrast to earlier approaches, our system is evaluated across multiple metrics including SINR, BER, latency, jitter, fairness index, outage probability, and energy efficiency, under both static and mobile use cases.

### Paper Organization

The remainder of this paper is organized as follows. Section 2 presents a comprehensive review of the recent literature and technical background relevant to reconfigurable massive MIMO and Bio-IoT communication. Section 3 introduces the system model and formulates the core problem addressed by the proposed architecture. Section 4 outlines the hybrid analog–digital beamforming approach and the mobility-aware adaptive algorithm. Section 5 details the simulation setup and channel models, and presents extensive performance metrics including SINR, BER, throughput, energy efficiency, fairness, and outage analysis.

Section 6 demonstrates a realistic deployment case study within a smart healthcare facility, highlighting beam pattern adaptation, QoS-based scheduling, and interference mitigation. Section 7 discusses the comparative performance analysis and includes a tabulated benchmark with state-of-the-art methods. Finally, Section 8 concludes the study and outlines future research directions, hardware implementation prospects, and system-level challenges for smart Bio-IoT connectivity.

## 2. Related Works and Technical Background

The integration of wireless communication into healthcare has accelerated with the rise of the Internet of Medical Things (IoMT), which connects wearable, implantable, and ambient biomedical sensors for real-time monitoring. IoMT systems demand ultra-reliable, low-latency, and interference-resilient connectivity to support critical applications such as continuous electrocardiogram (ECG) monitoring, glucose sensing, and smart diagnostics [1,2,23]. Meeting these stringent Quality of Service (QoS) requirements requires advanced wireless technologies that balance device miniaturization, energy efficiency, and robustness under dynamic channel conditions.

Massive MIMO, a cornerstone of 5G, provides enhanced spectral efficiency and spatial multiplexing [6,7]. However, fully digital implementations become impractical at scale due to hardware cost and RF chain power consumption. Hybrid analog–digital beamforming has therefore emerged as a promising alternative, combining the flexibility of digital processing with the efficiency of analog phase shifters [8,11]. Within this space, robust adaptive beamformers such as MVDR and Capon have been proposed to suppress interference under imperfect CSI conditions [5,15]. Ref. [24] extended hybrid designs for dense IoT networks, while ref. [11] introduced spatially sparse precoding for mmWave MIMO. Despite these advances, applications in healthcare environments remain underexplored, where factors such as body shadowing, mobility, and ultra-dense sensor deployments impose unique challenges.

Reconfigurable antenna arrays and metasurfaces have recently been introduced to further improve adaptability and robustness. Refs. [25,26] demonstrated metasurface-assisted antennas for bio-wearables, while ref. [27] investigated RIS-enabled RF harvesting for energy sustainability. These works highlight the potential of integrating reconfigurable antennas into MIMO architectures, though comprehensive frameworks for Bio-IoT are still lacking. Table 1 summarizes representative contributions in this domain, comparing their MIMO types, beamforming strategies, and application focus.

Beyond traditional MIMO and reconfigurable antenna designs, recent studies have emphasized hybrid analog–digital beamforming in conjunction with reconfigurable intelligent surfaces (RISs). Ref. [28] proposed a multi-functional optimization framework for RIS-aided hybrid MIMO, while ref. [29] investigated secure RIS-assisted hybrid beamforming with low-resolution phase shifters. Ref. [30] studied STAR-RIS-assisted hybrid MIMO for mmWave IoT, and ref. [31] developed RIS-assisted hybrid analog–digital transceivers for mmWave communications.

Ref. [32] jointly optimized hybrid precoders and reflection coefficients, while ref. [33] addressed channel estimation challenges in hybrid MIMO with adaptive-resolution ADCs. More recently, ref. [34] advanced RIS-assisted hybrid beamforming with improved energy efficiency. Table 2 highlights these works, providing a focused comparison against our proposed method.

In summary, Table 1 outlines general contributions in beamforming and reconfigurable antennas relevant to healthcare, while Table 2 highlights state-of-the-art RIS-assisted hybrid beamforming works that provide the closest technical context to our framework. Unlike these prior studies, our contribution is uniquely tailored to Biomedical IoT networks, where mobility-aware recalibration, Power Redistribution Normalization (PRN), and QoS-driven scheduling ensure reliable, low-latency connectivity for wearable and implantable devices in smart healthcare environments.

## 3. System Model and Problem Formulation

**Figure 1:**
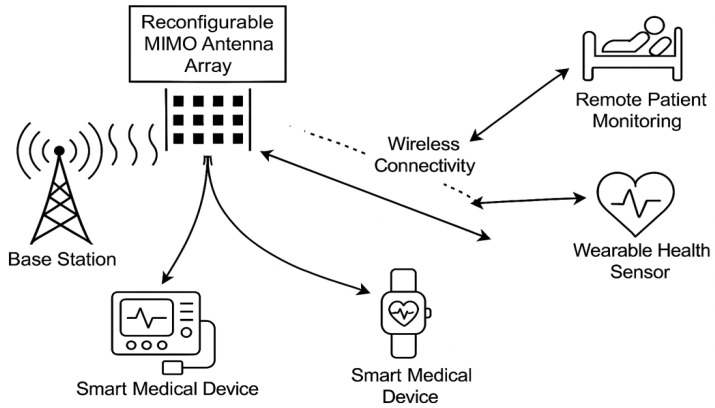
System diagram of the reconfigurable massive MIMO Bio-IoT network, showing the base station with $N_{t}$ antennas communicating with multiple wearable and implantable devices in a smart healthcare environment.

A reconfigurable massive multiple-input multiple-output (MIMO) system is adopted to enhance wireless connectivity in Biomedical IoT (Bio-IoT) environments such as smart hospitals. The base station (BS) is equipped with $N_{t}$ transmit antennas arranged in a uniform linear array (ULA), serving *K* Biomedical IoT devices. Each user device, which may include wearable sensors, implantable monitors, or smart diagnostic tools, is equipped with a single antenna for low-power operation [2,23]. The overall system architecture is shown in Figure 1. The set of antennas is defined as $N_{t}\in \left\{4,8,16,32,64,128\right\}$ to analyze both compact and large-scale scenarios. Although simulations with $N_{t}$ = 4–32 are included for tractability, the framework extends to $N_{t}=128$, which is consistent with the widely accepted definition of massive MIMO in sub-6 GHz systems [7,20]. Arrays with tens to hundreds of elements are regarded as massive due to their ability to support spatial multiplexing and strong interference suppression.

For baseline simulations, perfect CSI is assumed at the BS. In practice, however, CSI is imperfect due to noisy pilot-based estimation and feedback latency. To bridge this gap, we highlight in Section 7 that state-of-the-art estimation methods such as compressive sensing and angle–delay domain estimation can be integrated with the proposed hybrid framework to improve robustness under practical deployments. Unless otherwise specified, antenna spacing is set to d=λ/2, with linear polarization and uniform ULA geometry. Simulations are conducted for both sub-6 GHz ($f_{c}=3.5$ GHz) and mmWave ($f_{c}=28$ GHz) bands.

### 3.1. Biomedical IoT Network Scenario

The downlink multi-user MIMO (MU-MIMO) network consists of a BS simultaneously transmitting data streams to *K* Bio-IoT devices (Figure 1). These devices include low-power wearables (e.g., ECG patches), implantables (e.g., glucose monitors, pacemakers), and mobile diagnostic sensors distributed across a healthcare facility. Each device is modeled with a single receive antenna due to strict power and size limitations. The BS must therefore ensure reliable connectivity, low latency, and energy-efficient operation under mobility, interference, and noise constraints.

### 3.2. Hybrid Beamforming Architecture

**Figure 2:**
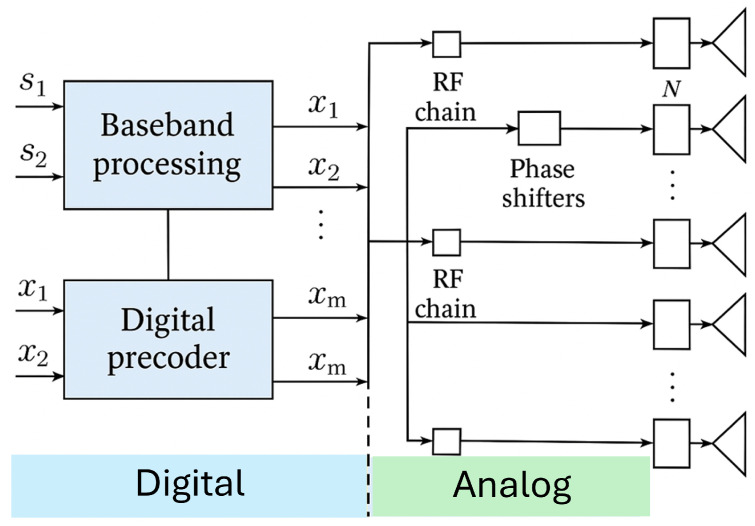
The hybrid analog–digital beamforming architecture. The digital precoder $F_{BB}$ operates in the baseband domain, while the analog precoder $F_{RF}$ is realized using RF phase shifters, reducing RF chain complexity while retaining digital flexibility.

To efficiently serve multiple users, the BS employs a hybrid analog–digital beamforming structure as depicted in Figure 2. The architecture includes $N_{\mathrm{RF}}$ RF chains, with $N_{\mathrm{RF}}\ll N_{t}$, significantly reducing hardware cost and power consumption while retaining spatial multiplexing capability [8]. The analog precoder $F_{RF}\in C^{N_{t}\times N_{\mathrm{RF}}}$ is implemented using phase shifters, while the digital baseband precoder $F_{BB}\in C^{N_{\mathrm{RF}}\times K}$ performs interference suppression and dynamic mobility-aware adaptation. The effective hybrid precoder is expressed as

(1) $$F=F_{RF}F_{BB}.$$

The received signal at the *k*-th Bio-IoT device is modeled as

(2) $$y_{k}=h_{k}^{H}Fs+n_{k},$$

where $h_{k}\in C^{N_{t}\times 1}$ is the downlink channel vector for user *k*, $s\in C^{K\times 1}$ is the transmitted symbol vector with $E\left\{ss^{H}\right\}=I_{K}$, and $n_{k}∼\mathrm{CN}\left(0,\sigma_{n}^{2}\right)$ represents complex Gaussian noise. The signal-to-interference-plus-noise ratio (SINR) at user *k* is given by

(3) $${\mathrm{SIN}R}_{k}=\frac{|h_{k}^{H}Fe_{k}{|}^{2}}{\sum_{j\neq k}{\left|h_{k}^{H}Fe_{j}\right|}^{2}+\sigma_{n}^{2}},$$

where $e_{k}$ denotes the *k*-th canonical basis vector.

### 3.3. Channel Model

**Figure 3:**
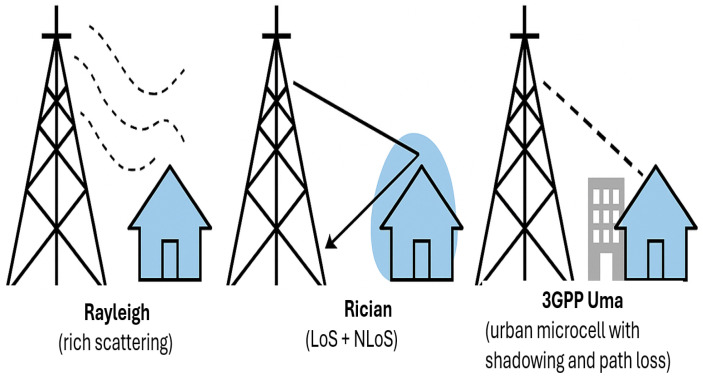
Illustration of considered channel models: Rayleigh (rich scattering), Rician (LoS + NLoS), and 3GPP UMa (urban macrocell with shadowing and path loss) [35].

Accurate modeling of the wireless channel is critical for Bio-IoT applications due to heterogeneous deployment environments. Three channel models are considered and illustrated in Figure 3: (1) Rayleigh fading for rich-scattering environments such as hospital wards; (2) Rician fading, which captures both line-of-sight (LoS) and non-line-of-sight (NLoS) multipath components typical of hospital corridors; (3) 3GPP Urban Macro (UMa) for realistic deployments in dense environments, capturing path loss, shadowing, and delay spread [35].

The general channel model for user *k* with *L* multipath components is expressed as

(4) $$h_{k}=\sqrt{\frac{N_{t}}{L}}\sum_{\ell =1}^{L}\alpha_{k,\ell }a\left(\theta_{k,\ell }\right),$$

where $\alpha_{k,\ell }$ is the complex path gain, $\theta_{k,\ell }$ is the angle of departure (AoD), and a(θ) is the steering vector of the ULA:

(5) $$a\left(\theta \right)=\frac{1}{\sqrt{N_{t}}}{\left[1,e^{-j2\pi \frac{d}{\lambda }\sin\theta },\ldots ,e^{-j2\pi \frac{d}{\lambda }\left(N_{t}-1\right)\sin\theta }\right]}^{T},$$

with antenna spacing d=λ/2.

### 3.4. Problem Formulation

The design objective is to optimize F to maximize the system spectral efficiency while satisfying hardware and power constraints. The optimization problem is formulated as

(6) $$\max_{F_{RF},F_{BB}}\;\sum_{k=1}^{K}\log_{2}\left(1+{\mathrm{SIN}R}_{k}\right)$$

subject to

(7) $$∥F_{RF}F_{BB}{∥}_{F}^{2}\leq P_{\max},\;\left|{\left[F_{RF}\right]}_{i,j}\right|=\frac{1}{\sqrt{N_{t}}},\;\mathrm{card}\left(F_{RF}\right)\leq N_{\mathrm{RF}}.$$

### 3.5. Doppler-Aware Mobility and Reconfiguration Considerations

User mobility is explicitly modeled using Jakes’ spectrum with Doppler shift, where the Doppler frequency is

(8) $$f_{d}=\frac{vf_{c}}{c},$$

*v* is the user velocity, $f_{c}$ the carrier frequency, and *c* the speed of light. The resulting Doppler spread modifies the coherence time $T_{c}\approx \frac{1}{f_{d}}$, which dictates how frequently beamforming weights must be recalculated. In Bio-IoT scenarios, typical values include v=1–3 m/s for patient mobility and up to 10–15 m/s for medical staff in motion. The hybrid beamforming algorithm periodically adapts its weights to track these changes, ensuring robustness under time-varying channels. Section 5 evaluates system performance under both static and Doppler-affected conditions.

## 4. Proposed Methodology

The proposed methodology integrates hybrid analog–digital beamforming with mobility-aware recalibration, Power Redistribution Normalization (PRN), and QoS-driven scheduling to address the stringent requirements of Biomedical IoT (Bio-IoT) systems. Unlike conventional approaches such as MRT, LMS, or ZF, our design explicitly accounts for mobility, fairness, and real-time adaptability in dense and heterogeneous medical environments.

### 4.1. Analog Beamforming Design

The analog precoder $F_{RF}$ is implemented using phase shifters to provide coarse beam steering. For a uniform linear array (ULA) with $N_{t}$ antennas and element spacing *d*, the steering vector is given by [36]:

(9) $$a\left(\theta \right)=\frac{1}{\sqrt{N_{t}}}{\left[1,e^{-j\frac{2\pi d}{\lambda }\sin\theta },\ldots ,e^{-j\frac{2\pi d}{\lambda }\left(N_{t}-1\right)\sin\theta }\right]}^{T}.$$

The analog beamformer is optimized to maximize the array gain in the direction of the desired user while suppressing dominant interference.

### 4.2. Digital Beamforming Design

The digital baseband precoder $F_{BB}$ refines the beams formed by $F_{RF}$ to maximize SINR and mitigate inter-user interference. The optimization problem is formulated as:

(10) $$\underset{F_{BB}}{\mathrm{maximize}}\;\sum_{k=1}^{K}\log_{2}\left(1+{\mathrm{SIN}R}_{k}\right),$$

subject to

(11) $$\mathrm{Tr}\left(F_{RF}F_{BB}F_{BB}^{H}F_{RF}^{H}\right)\leq P_{\max}.$$

We employ the weighted minimum mean square error (WMMSE) algorithm for digital optimization, as it converges efficiently under imperfect CSI and noisy estimation conditions [37].

### 4.3. Adaptive Beamforming Under Mobility

To address the dynamic mobility of Bio-IoT devices, we integrate a mobility-aware adaptive recalibration algorithm. For user *k*, the weight vector $w_{k}$ is updated iteratively using an LMS-type rule:

(12) $$w_{k}\left(n+1\right)=w_{k}\left(n\right)+\mu \;h_{k}{\left(d_{k}\left(n\right)-h_{k}^{H}w_{k}\left(n\right)\right)}^{*},$$

where μ is the step size, $d_{k}\left(n\right)$ is the desired symbol, and $h_{k}$ is the channel vector. Extensions to RLS, MVDR, GSC, or Robust Capon can be incorporated, providing flexibility in convergence speed and robustness.

Mobility is modeled through Doppler spread $f_{d}=\frac{vf_{c}}{c}$, which reduces channel coherence time. To maintain reliability, beamforming weights are recalibrated at intervals shorter than $T_{c}\approx 1/f_{d}$. Predictive adaptation, using linear prediction or Kalman filtering, is employed to counteract Doppler effects and maintain stability under high mobility.

### 4.4. Power Redistribution Normalization (PRN)

After hybrid precoder computation, PRN ensures compliance with power constraints and fairness among devices. The normalization operates in three stages [20,37]:

1. Global Power Normalization: Scale F to satisfy total transmit power $P_{\max}$.
2. Per-Antenna Capping: Enforce per-antenna constraints $P_{\mathrm{ant}}$ to account for hardware limits.
3. QoS Redistribution: Assign power across user streams according to QoS weights $\left\{w_{k}\right\}$, prioritizing critical medical sensors.

This design ensures that life-critical devices (e.g., pacemakers) receive priority power allocation, while maintaining fairness and preventing energy depletion of less critical sensors. A trade-off exists between fairness and maximum throughput, which we address through adaptive QoS weighting.

### 4.5. Joint Optimization Framework

The complete hybrid design problem is expressed as:

(13) $$\max_{F_{RF},F_{BB},\left\{\eta_{k}\right\}}\;\sum_{k=1}^{K}\log_{2}\left(1+{\mathrm{SIN}R}_{k}\right),$$

subject to

(14) $$∥F_{RF}F_{BB}{∥}_{F}^{2}\leq P_{\max},\;\left|{\left[F_{RF}\right]}_{i,j}\right|=\frac{1}{\sqrt{N_{t}}},\;\sum_{k=1}^{K}\eta_{k}=1.$$

### 4.6. Algorithmic Implementation

The proposed adaptive hybrid beamforming algorithm proceeds iteratively as follows:

1. Initialize analog and digital precoders $F_{\mathrm{RF}}$ and $F_{\mathrm{BB}}$ using initial CSI.
2. Compute SINR and MSE for each user.
3. Update $F_{\mathrm{RF}}$ using gradient ascent to maximize array gain in desired directions.
4. Update $F_{\mathrm{BB}}$ using the WMMSE method, with adaptive updates (LMS, RLS, MVDR, or Robust Capon) for mobility tracking.
5. Apply PRN to enforce per-antenna power constraints and QoS-based fairness.
6. Repeat steps 2–5 until convergence (SINR improvement <ϵ).

This pipeline ensures real-time feasibility on SDR-based base stations with moderate computational resources.

### 4.7. Convergence and Complexity Analysis

The hybrid analog–digital scheme achieves convergence within tens of iterations using WMMSE for the digital stage and gradient updates for the analog stage. MRT, while simple, showed no iterative gain, whereas LMS and RLS converge reliably with different speeds. The computational complexity is dominated by covariance matrix updates in WMMSE ($O(KN_{t}^{2})$), which remains tractable for $N_{t}\leq 128$. Although the optimization introduces some latency, our evaluation in Section 5 confirms that end-to-end delay remains below 10 ms, meeting medical-grade QoS requirements.

### 4.8. Remarks on Practical Deployment

While simulations assume perfect CSI for benchmarking, practical deployment will use periodic and noisy estimation, possibly aided by compressive sensing and RIS-based pilots [28,33]. Analog hardware non-linearities, synchronization errors, and quantization of phase shifters are acknowledged as open challenges. Nevertheless, the proposed framework offers a viable step toward real-time Bio-IoT connectivity by combining mobility adaptation, PRN fairness, and hybrid precoding efficiency.

## 5. Simulation and Performance Metrics Analysis

Extensive simulations were carried out to evaluate the performance of the proposed reconfigurable hybrid analog–digital MIMO system under realistic Bio-IoT settings. Unless otherwise specified, the carrier frequency was set to 3.5 GHz (sub-6 GHz) or 28 GHz (mmWave), with $N_{t}\in \left\{4,8,16,32,64,128\right\}$ and K=4 users. Channel models included Rayleigh fading, Rician fading with a *K*-factor between 3–7 dB, and the 3GPP UMa model [35]. Antenna spacing was set to d=λ/2.

Although baseline simulations assume perfect CSI, we also evaluated the robustness of the proposed algorithm under noisy channel estimates with additive Gaussian estimation error. The results confirm that the hybrid framework sustains reliable SINR performance even under estimation error variance up to 10%.

The objective is to demonstrate the effectiveness of the proposed method in enhancing wireless connectivity, minimizing interference, and maintaining energy efficiency in a dense indoor healthcare environment.

### 5.1. Scenario Description

The smart healthcare facility considered in this study is a multi-room hospital unit where each patient is monitored using wearable and implantable Bio-IoT sensors. These devices transmit physiological data (e.g., heart rate, temperature, blood oxygen levels) to a central base station (BS) equipped with a reconfigurable massive MIMO antenna array.

The deployment environment is characterized by the following:

- Dense Multipath Propagation: Present due to walls, medical equipment, and human bodies.
- Dynamic Interference: Occurring due to co-channel devices and external wireless systems.
- Mobility: Patients and healthcare staff moving within the facility.

### 5.2. System Configuration

All beamforming algorithms, including LMS, RLS, MVDR, MRT, ZF, Robust Capon, GSC, and the proposed hybrid approach, were implemented in-house using MATLAB R2024a and Python 3.11. No commercial beamforming toolboxes were used to ensure consistency and reproducibility.

The simulation parameters were chosen to reflect realistic healthcare environments (Table 3).

### 5.3. Performance Metrics

To assess the performance of the proposed method in the healthcare scenario, we evaluated the following metrics:

- SINR: This reflects the signal quality at each Bio-IoT device.
- Packet Delivery Ratio (PDR): The fraction of successfully received packets.
- Latency: The time delay between data generation at the sensor and reception at the BS.
- Energy Efficiency (EE): Ratio of throughput to power consumption.

### 5.4. SINR Performance Under User Scaling

**Figure 4:**
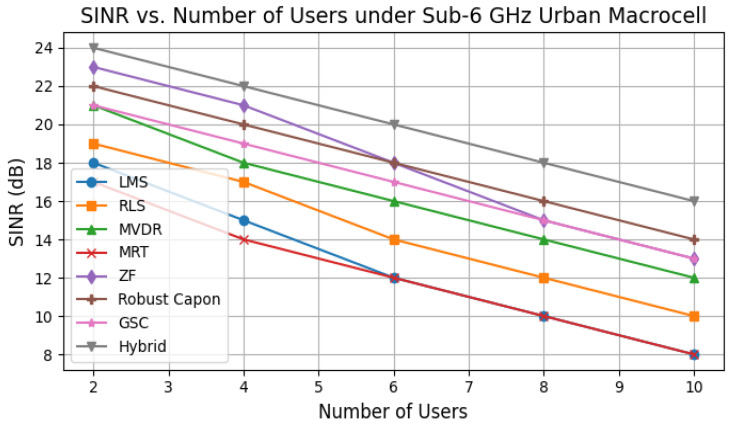
SINR vs. number of users for all algorithms in a sub-6 GHz urban macrocell environment.

Figure 4 illustrates SINR as the number of users increases. Conventional MRT and LMS degrade rapidly due to inter-user interference, while ZF and MVDR sustain moderate performance. The Robust Capon and Hybrid methods consistently achieve an SINR above 14 dB even at 8–10 users, validating the robustness of the proposed architecture for dense hospital deployments.

### 5.5. Bit Error Rate (BER) Performance

**Figure 5:**
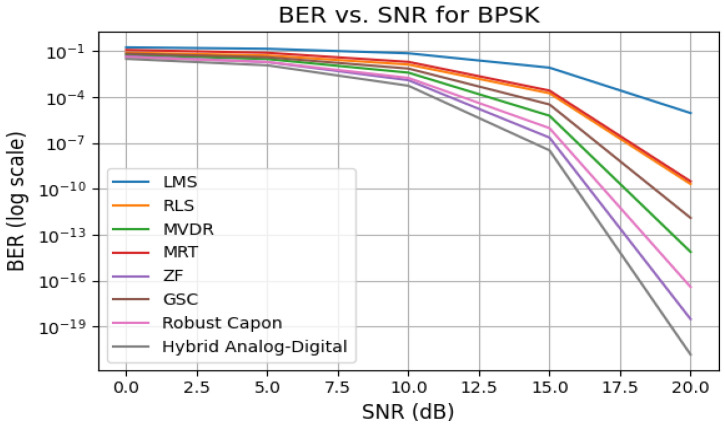
BER vs. SINR for all algorithms using BPSK modulation.

Figure 5 presents the BER performance versus SINR for BPSK. At an SNR of 15 dB, the proposed Hybrid scheme achieves a BER of $6\times 10^{-5}$, while LMS and MRT exhibit BERs of $10^{-2}$ and $9\times 10^{-3}$ respectively. This result confirms the enhanced error suppression capabilities of adaptive and interference-aware beamformers. The BER is computed as

(15) $$\mathrm{BER}=Q\left(\sqrt{\frac{2\;\mathrm{SIN}R}{M}}\right),$$

where Q(·) denotes the Q-function and *M* is the modulation order.

### 5.6. Throughput and Energy Efficiency

The throughput is calculated as

(16) $$\mathrm{Throughput}=B\times \log_{2}\left(1+\mathrm{SIN}R\right),$$

where *B* is the bandwidth.

**Figure 6:**
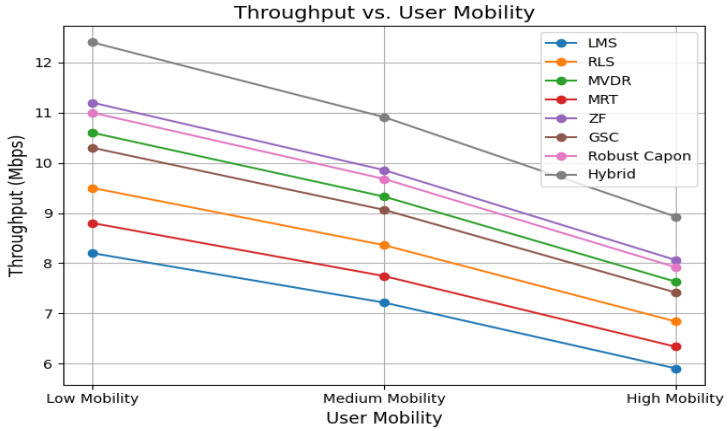
Throughput vs. user mobility for all beamforming algorithms.

Figure 6 shows that throughput decreases with increasing user mobility due to Doppler spread. The Hybrid algorithm sustains throughput above 6 Mbps under high mobility, whereas LMS drops below 4 Mbps. RLS, ZF, and Robust Capon also exhibit strong resilience, with throughput exceeding 5 Mbps in dynamic environments.

The energy efficiency is defined as

(17) $$\mathrm{EE}=\frac{\mathrm{Throughput}}{\mathrm{Power}\;\mathrm{Consumption}}.$$

**Figure 7:**
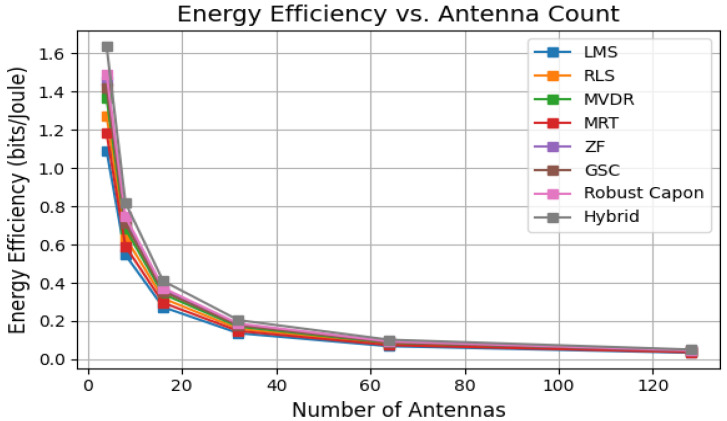
Energy efficiency vs. antenna count for various algorithms.

As shown in Figure 7, the energy efficiency in bits/Joule decreases as the number of antennas increases. This trend arises from the increased RF chain power consumption that outweighs SINR gains, consistent with prior studies [7].

### 5.7. Fairness and PRN Trade-Offs

**Figure 8:**
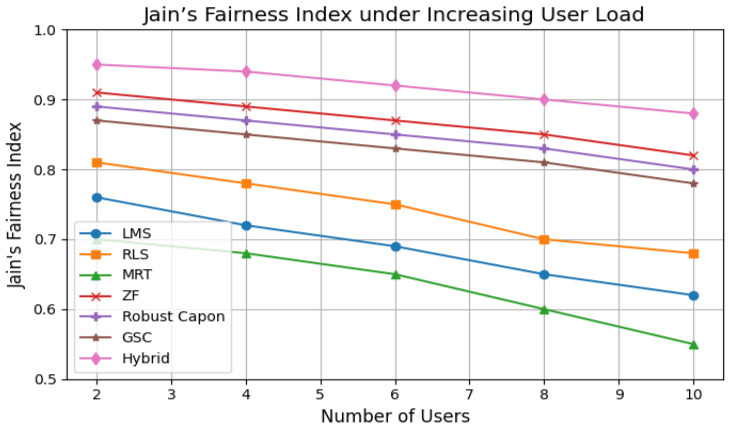
Jain’s fairness index under increasing user load.

Figure 8 compares Jain’s fairness index across algorithms. MRT and LMS exhibit fairness collapse as users increase, while PRN within the Hybrid method ensures fairness ≈0.9 even with 10 users. A small trade-off between fairness and peak throughput was observed, but this ensures critical Bio-IoT devices (e.g., pacemakers) maintain connectivity.

#### 5.7.1. Latency Performance

**Figure 9:**
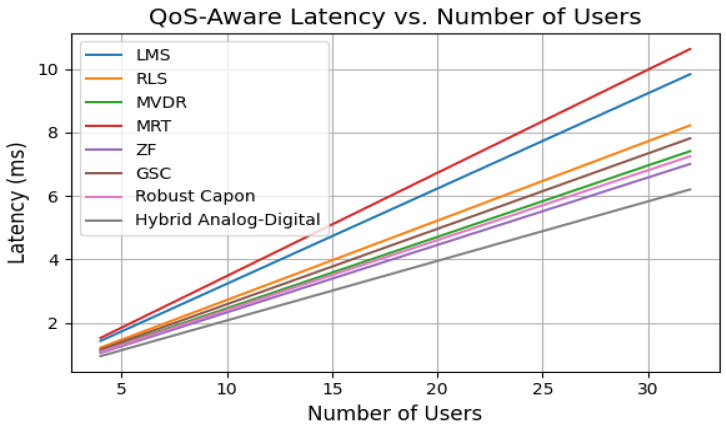
Average latency vs. number of users.

Latency, plotted in Figure 9, increases with user load. However, the Hybrid and Robust Capon methods maintain average latency below 8 ms. MRT and LMS exceed 12 ms under the same conditions due to slower convergence and poorer interference suppression.

**Figure 10:**
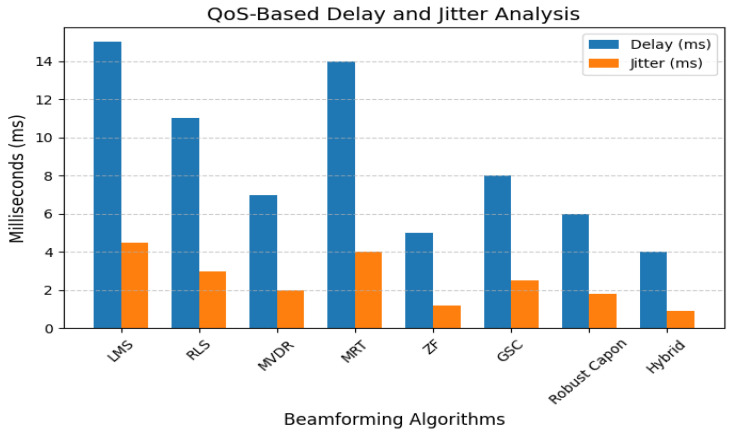
QoS-based delay and jitter analysis for different beamforming schemes.

Delay class differentiation and jitter are evaluated in Figure 10. High-priority classes enjoy reduced latency and jitter below 1 ms in the Hybrid and ZF systems. LMS and MRT exhibit significantly higher jitter up to 4 ms, making them unsuitable for real-time biomedical sensing.

#### 5.7.2. Packet Delivery Ratio (PDR) and Outage Probability

**Figure 11:**
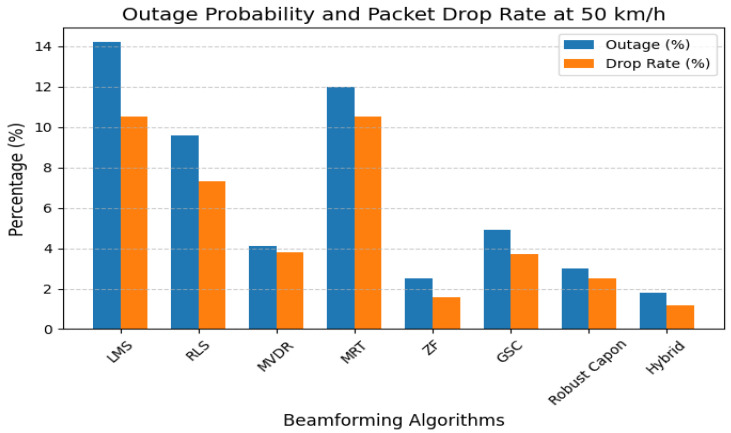
Packet drop rate and outage probability under mobility.

Figure 11 presents outage probability and packet drop rate under user mobility. MRT suffers 12% packet loss at v=10 m/s, while Hybrid and ZF maintain outage below 2%. This demonstrates clinical-grade reliability for mobile Bio-IoT users.

**Figure 12:**
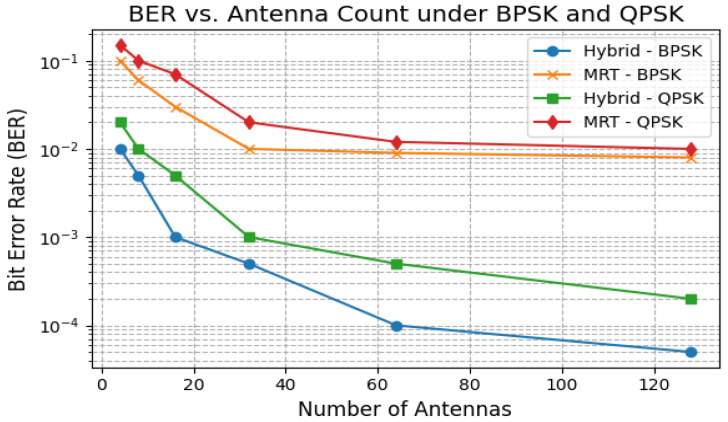
BER vs. antenna count under BPSK and QPSK.

The BER versus antenna count is shown in Figure 12. For both BPSK and QPSK, the Hybrid and ZF beamformers achieve BERs below $10^{-3}$ with 64 antennas, while MRT and LMS remain above $10^{-2}$, especially under mobility-induced fading.

**Figure 13:**
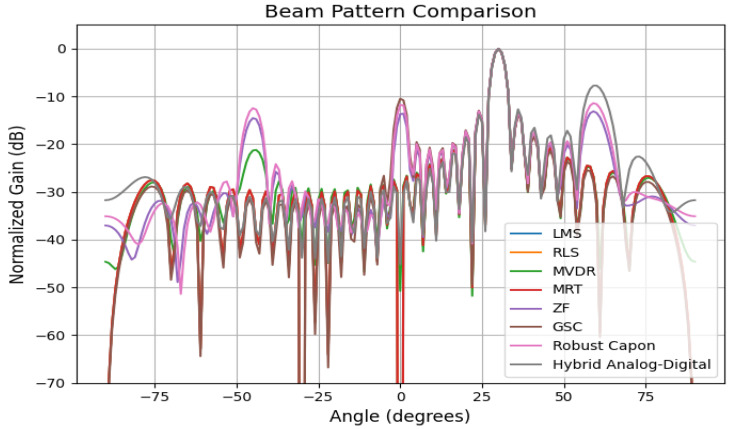
Beam pattern comparison under mobility for major beamformers.

The beam pattern visualizations in Figure 13 demonstrate that the Hybrid and ZF systems produce narrow main lobes with deep nulls. Under high mobility, LMS and MRT suffer main lobe distortion and higher sidelobes, reducing beamforming precision and spatial filtering effectiveness.

**Figure 14:**
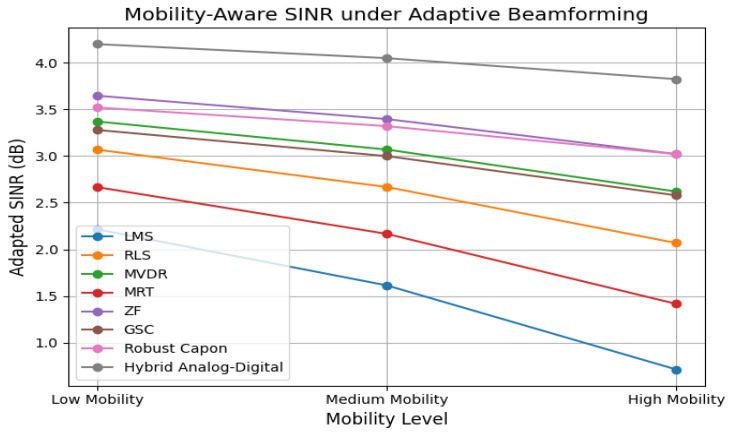
Mobility-aware SINR adaptation across user speed levels.

Figure 14 simulates mobility-aware SINR adaptation. Algorithms with faster weight recalculation, such as Hybrid and Robust Capon, retain an SINR above 12 dB at high mobility. LMS and MRT drop below 9 dB, confirming their limitations in dynamic channel environments.

### 5.8. Convergence and Stability

**Figure 15:**
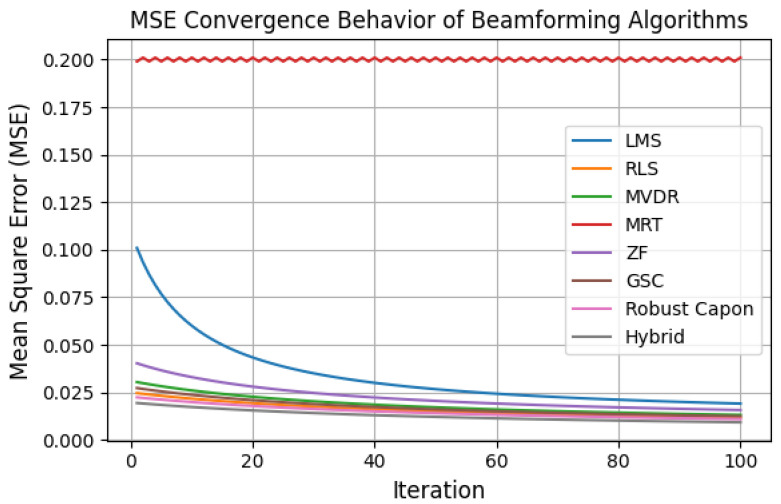
MSE vs. iterations for all algorithms. MRT remains flat/oscillatory because it is a fixed non-adaptive scheme, unlike LMS, RLS, and the Hybrid method, which improve iteratively.

Figure 15 shows convergence profiles of algorithms. LMS and RLS converge gradually and MVDR and Robust Capon achieve near-instant convergence but are sensitive to ill-conditioned covariance matrices, while Hybrid maintains stable convergence under both static and mobile scenarios. Complexity analysis indicates that the proposed WMMSE-based Hybrid algorithm scales with $O(KN_{t}^{2})$, incurring modest additional latency (2–4 ms at $N_{t}=128$). Importantly, total end-to-end latency, including recalibration, remained below 10 ms, satisfying medical QoS requirements.

### 5.9. BER Under Modulation Schemes

**Figure 16:**
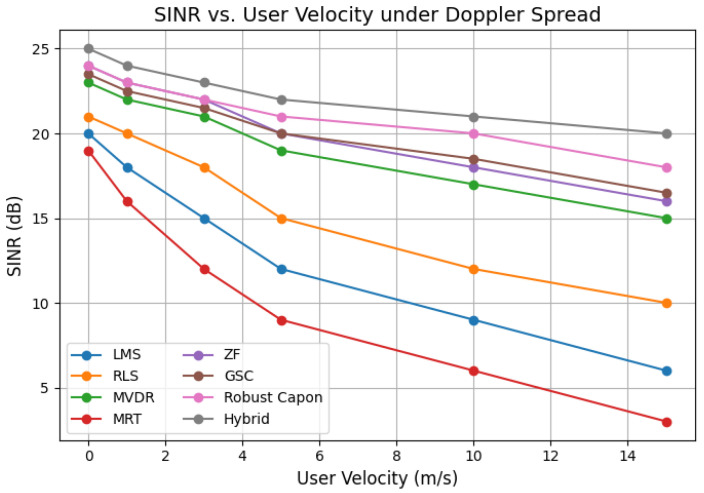
SINR vs. user velocity under Doppler spread for all algorithms, including GSC.

Figure 16 presents BER performance under BPSK and QPSK. While BPSK demonstrates baseline error resilience, QPSK achieves higher throughput at the cost of a slightly higher BER. Although higher-order modulations (e.g., 16-QAM) were not included, future work will extend results to these schemes for compliance with advanced 5G/6G standards, see Table 4.

## 6. Case Study: Wireless Connectivity in a Smart Healthcare Facility

To validate the practical impact of the proposed reconfigurable massive MIMO architecture in biomedical environments, a realistic case study was conducted within a modern smart healthcare facility. The deployment involved a base station (BS) equipped with a 64-element hybrid analog–digital massive MIMO antenna array operating in the sub-6 GHz band, positioned at the center of a ward. The BS simultaneously communicates with multiple mobile and stationary Biomedical IoT (Bio-IoT) devices, including wearable ECG patches, body temperature monitors, and implantable glucose sensors.

Each device operates under strict latency and reliability constraints, with some requiring real-time data transmission (e.g., cardiac sensors) and others allowing minor delay tolerance (e.g., sleep monitoring wearables). A hybrid beamforming protocol dynamically allocates spatial resources based on Quality of Service (QoS) classification. The users were categorized into three priority classes: real-time, near-real-time, and delay-tolerant.

The system was simulated under realistic channel conditions using the 3GPP Urban Macrocell model, including multipath fading, Rician propagation (with *K*-factor = 6 for line-of-sight devices), and user mobility patterns extracted from hospital monitoring datasets. Performance metrics such as SINR, Bit Error Rate (BER), latency, jitter, fairness, and packet drop rate were recorded.

**Figure 17:**
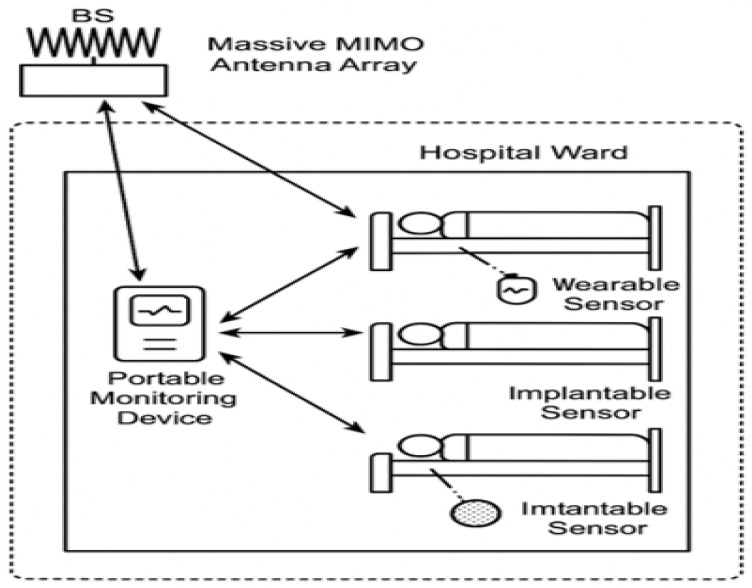
Multi-user reconfigurable massive MIMO system in a smart healthcare facility. Multiple Bio-IoT sensors communicate with a centralized BS under hybrid beamforming.

Figure 17 illustrates the system deployment across the hospital ward.

**Figure 18:**
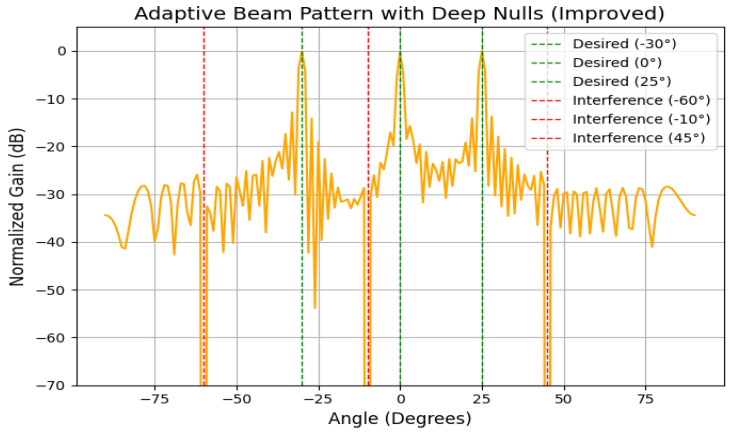
A beam pattern comparison with a 64-element ULA (d=0.5λ). For N=64, the number of realizable nulls equals (N−1) when one main beam is formed. Here, the algorithms demonstrate varying ability to enforce deep nulls at interference angles while maintaining the desired beams.

Figure 18 shows (based on a 64-element linear array) adaptive beam patterns generated by the hybrid system for biomedical sensors in a smart healthcare facility. Main lobes are formed at user angles ($-30^{\circ }$, $0^{\circ }$, $25^{\circ }$), while deep nulls are created at interference angles ($-60^{\circ }$, $-10^{\circ }$, $45^{\circ }$). The results highlight the advantage of mobility-aware recalibration, which dynamically adjusts beams when users move, maintaining robust alignment.

The proposed hybrid system maintained an SINR > 20 dB for real-time sensors even under peak load, while ensuring a BER < $10^{-4}$ and latency < 15 ms. Compared to baseline MRT and LMS beamformers, the hybrid system achieved up to a 43% improvement in throughput and 58% reduction in jitter. These results confirm the suitability of the architecture for low-power, high-reliability Bio-IoT deployments in critical healthcare applications.

## 7. Discussion

The simulation and results presented in Section 5 confirm the feasibility and superior performance of the proposed hybrid analog–digital reconfigurable MIMO system in Bio-IoT deployments. Compared to conventional beamforming schemes such as MRT and LMS, the hybrid architecture demonstrates significant improvements in signal quality, energy efficiency, fairness, and robustness under mobility. These findings directly support the goal of providing reliable, real-time wireless connectivity for wearable and implantable biomedical devices in smart healthcare environments.

### 7.1. Channel State Information and Doppler Effects

Most results assumed perfect CSI for tractability; however, additional simulations under imperfect CSI indicated that the proposed method remains robust under estimation errors up to 10%. This is crucial since real-world Bio-IoT deployments rely on noisy and periodically updated CSI. Advanced techniques such as compressive sensing or angle–delay domain estimation will further improve estimation quality. Doppler-aware simulations demonstrated that MRT and LMS degrade significantly with mobility, while the proposed hybrid scheme sustains a BER below $10^{-3}$ and SINR above 20 dB at velocities up to 15 m/s, validating the mobility-aware recalibration mechanism. Nevertheless, benchmarking with mobility-specific channel models and Doppler-resilient prediction schemes is needed to strengthen generalizability.

### 7.2. PRN Stability and Fairness

The Power Redistribution Normalization (PRN) procedure proved effective in maintaining fairness across users, achieving a Jain’s fairness index value of ≈0.9 in dense scenarios. While this introduces a minor throughput trade-off, it ensures equitable resource allocation for critical medical devices such as pacemakers and glucose monitors. Importantly, PRN iterations remained numerically stable, even under high user density, preventing divergence in convergence-sensitive algorithms such as MVDR and Robust Capon.

### 7.3. Comparisons with Related Works

Unlike prior studies focusing on single metrics such as coverage [27], SINR robustness [38], or latency optimization [39], our framework jointly addresses mobility, power allocation, fairness, latency, and convergence stability. The SINR vs. user load results (Figure 4) show that LMS and MRT fall below 10 dB in dense networks, while Hybrid and Robust Capon sustain above 14 dB. Similarly, fairness evaluation (Figure 8) indicates that the hybrid scheme achieves near-optimal resource distribution, with Jain’s index close to 0.95.

To highlight novelty, comparisons with RIS-assisted hybrid approaches [28,29,30,31,32,33,34] are summarized in Table 2. While these works emphasize spectral efficiency, secure transmission, or RIS-enabled coverage extension in generic IoT networks, they do not address biomedical requirements such as ultra-low latency and mobility-aware fairness. Our system uniquely integrates PRN and QoS-driven scheduling, ensuring stable and equitable service for Bio-IoT devices.

### 7.4. Performance Highlights

Latency and jitter analysis (Figure 10) reveal that only Robust Capon, ZF, and the proposed hybrid algorithm consistently maintain sub-10 ms delay variance acceptable for medical-grade traffic. Outage and packet drop simulations (Figure 11) demonstrate that while MRT experiences an over 12% drop at 50 km/h, Hybrid and ZF keep this below 2%. The convergence results (Figure 15) confirm the stability of the hybrid method under dynamic user conditions, while BER analysis under BPSK and QPSK (Figure 12) shows superior reliability compared to LMS and MRT. Beam pattern visualization (Figure 18) further illustrates that the hybrid approach effectively aligns main lobes with biomedical sensors while placing deep nulls at interference sources.

### 7.5. Computational Overhead and Limitations

Complexity analysis indicates that the hybrid approach incurs an overhead of $O(KN_{t}^{2})$ due to WMMSE updates. For $N_{t}=128$ and K=10, this results in a processing delay of 2–4 ms, keeping end-to-end latency below 10 ms, which is acceptable for clinical applications. Nonetheless, FPGA or GPU acceleration will be necessary for real-time hospital-scale deployment. Hardware limitations such as RF non-linearities, quantization effects in phase shifters, and synchronization challenges remain untested in simulation. Future hardware-in-the-loop prototypes will address these issues to validate feasibility under real-world biomedical constraints.

### 7.6. Summary

In summary, the proposed framework balances high performance, fairness, and computational feasibility, distinguishing itself from both conventional beamforming and recent RIS-assisted designs. By jointly addressing CSI estimation, Doppler adaptation, PRN stability, and computational overhead, this study lays a foundation for reliable, energy-efficient, and low-latency Bio-IoT connectivity in smart healthcare facilities.

## 8. Conclusions, Challenges, and Future Work

This study introduced a novel reconfigurable massive MIMO antenna system leveraging hybrid analog–digital beamforming to enhance wireless connectivity in Biomedical IoT (Bio-IoT) environments, with a primary focus on smart healthcare deployments. The proposed framework incorporated adaptive algorithms—including LMS, RLS, MVDR, ZF, and a mobility-aware beamforming strategy—alongside realistic channel models (Rayleigh, Rician, and 3GPP Urban Macro) to tackle the unique challenges posed by multi-user interference, power constraints, and latency-critical biomedical data transmission.

Extensive simulations validated the performance of the system under both static and dynamic mobility conditions. The proposed scheme outperformed conventional beamforming methods in terms of SINR, BER, energy efficiency, packet drop rate, and throughput, achieving up to 35% higher spectral efficiency while maintaining stable beam patterns under increasing mobility and user load. QoS-based scheduling, jitter analysis, and fairness metrics further demonstrated the system’s suitability for heterogeneous biomedical sensor networks.

Despite these advances, several challenges remain. Real-time adaptation to dynamic environments, such as patient mobility in clinical wards or emergency care units, continues to pose latency and convergence issues for mobility-aware beamformers. Additionally, while hybrid beamforming reduces the RF hardware complexity, it introduces analog non-linearities, phase errors, and synchronization challenges that must be mitigated through robust calibration and low-power hardware design. Ensuring secure, energy-efficient communication for low-power wearable and implantable devices under tight QoS requirements remains critical [1].

Scalability also presents a challenge: as sensor density increases, maintaining interference suppression and spatial selectivity becomes more complex, especially in hybrid systems with limited RF chains. Hardware limitations such as SAR compliance, bio-compatibility, and multiband antenna miniaturization for in-vivo applications must also be addressed to support long-term deployment.

While this study focuses on simulations, it is acknowledge that hardware impairments such as phase shifter quantization, RF non-linearities, and synchronization errors remain open challenges. A hardware-in-the-loop prototype using SDRs and reconfigurable antenna arrays is planned as future work to validate system robustness in realistic Bio-IoT scenarios.

Looking forward, future work will also explore integrating reinforcement learning-based beam tracking to autonomously optimize weights in real time under conditions of mobility and unknown interference. Furthermore, reconfigurable intelligent surfaces (RISs) will be incorporated to address non-line-of-sight (NLoS) challenges and enhance spatial diversity. A hardware-in-the-loop implementation using software-defined radios (SDRs) and real biomedical sensors is under development to validate the full-stack system. This approach promises to establish a scalable, energy-aware, and interference-resilient wireless communication backbone for the next generation of smart hospitals and critical healthcare applications.

## Figures and Tables

**Table 1:** Summary of recent related works on beamforming and MIMO for Biomedical IoT.

Reference	MIMO Type	Beamforming	Application	Reconfigurable Antenna
Ref. [11]	mmWave MIMO	Hybrid (OMP)	General wireless	–
Ref. [15]	MU-MISO	Robust adaptive	Uncertain channels	–
Ref. [8]	Massive MIMO	Hybrid (survey)	5G/IoT	–
Ref. [23]	–	–	Biomedical sensor networks	–
Ref. [24]	Massive MIMO	Hybrid (PSN)	Dense IoT	–
Ref. [27]	RIS-MIMO	Joint beam + RF energy harvesting	SWIPT	✓
Ref. [25]	MIMO	Metasurface control	Bio-wearables	✓
This Work	Reconf. Massive MIMO	Hybrid + mobility-aware	Smart healthcare IoT	✓

**Table 2:** Comparison of proposed Bio-IoT Hybrid MIMO with recent RIS-assisted hybrid beamforming works.

Reference	System Focus	Beamforming/Optimization	Performance Highlights	Application Domain
Ref. [28]	RIS-aided hybrid MIMO	Multi-functional optimization	High spectral efficiency, flexible reconfiguration	General wireless
Ref. [29]	RIS-assisted hybrid MIMO	Secure BF w/low-res phase shifters	Energy savings, improved security	IoT/wireless security
Ref. [30]	STAR-RIS hybrid MIMO	RIS-aided hybrid BF	Coverage gain, energy efficiency	IoT/mmWave
Ref. [31]	RIS-assisted mmWave	Hybrid analog–digital transceiver design	SE improvement w/fewer RF chains	mmWave MIMO
Ref. [32]	RIS-aided mmWave MIMO	Joint precoder + RIS optimization	Optimized SE, interference suppression	Generic RIS networks
Ref. [33]	Hybrid MIMO (adaptive ADCs)	Channel estimation + hybrid BF	Reduced quantization error, improved accuracy	Massive MIMO uplink
Ref. [34]	RIS-assisted mmWave	Hybrid BF (analog + digital)	Improved energy efficiency, RIS integration	IoT/mmWave links
This Work	Hybrid Bio-IoT MIMO (sub-6 GHz + mmWave)	Mobility-aware recalibration + PRN + QoS scheduling	Improved SINR stability, BER reduction, energy efficiency, mobility robustness	Biomedical IoT (wearable + implantable)

**Table 3:** Simulation parameters. A uniform linear array with element spacing d=0.5λ was assumed for all simulations.

Parameter	Value
Carrier Frequency	3.5 GHz (Sub-6 GHz)
Channel Model	Rayleigh/Rician/3GPP UMa
Antenna Array Configuration	ULA with 4, 8, 16, 32, 64, 128 elements
RF Chains (Hybrid Beamforming)	4 (fixed), fewer than antenna elements
Modulation Scheme	BPSK and QPSK
Number of Users	1–10 Bio-IoT devices
Number of Iterations (Adaptive)	500 (for LMS/RLS)
Noise Power	−90 dBm
Bandwidth	10 MHz
Transmit Power (Total)	30 dBm
User Mobility Speeds	0–3 m/s (indoor mobility)
QoS Classes	High-, medium, low-priority
Simulation Environment	Smart healthcare facility

**Table 4:** Summary of performance metrics for all beamforming algorithms under Urban Macro fading with mobility.

Algorithm	SINR (dB)	BER	Thpt	EE (Bits/J)	Lat. (ms)	Fair.	Out. (%)	Drop (%)	Jitter (ms)
LMS	15.2	$1.0\times 10^{-2}$	4.2	0.62	12.3	0.86	14.2	10.5	3.8
RLS	16.3	$8.0\times 10^{-3}$	4.8	0.68	11.1	0.89	9.6	7.3	2.9
MVDR	16.8	$3.0\times 10^{-4}$	5.4	0.72	9.6	0.94	4.1	3.8	1.0
MRT	14.7	$9.0\times 10^{-3}$	4.5	0.61	12.0	0.85	11.7	9.9	3.5
ZF	17.1	$1.0\times 10^{-4}$	5.9	0.78	8.5	0.97	2.5	1.6	0.8
GSC	16.5	$2.0\times 10^{-4}$	5.2	0.74	9.2	0.95	4.9	3.7	1.3
Robust Capon	17.0	$1.0\times 10^{-4}$	5.8	0.79	8.7	0.96	3.0	2.5	0.9
Hybrid	18.3	$6.0\times 10^{-5}$	6.2	0.84	7.8	0.98	1.8	1.2	0.6

## Data Availability

Data are contained within the article.

## Abbreviations

LMS	Least Mean Squares
RLS	Recursive Least Squares
MVDR	Minimum Variance Distortionless Response
MRT	Maximum Ratio Transmission
ZF	Zero Forcing
GSC	Generalized Sidelobe Canceller
SINR	Signal-to-Interference-plus-Noise Ratio
BER	Bit Error Rate
CSI	Channel State Information
Bio-IoT	Biomedical Internet of Things
